# Reduced Cacna1c Expression Produces Anhedonic Reactions to Palatable Sucrose in Rats: No Interactions With Juvenile or Adult Stress

**DOI:** 10.1111/gbb.70021

**Published:** 2025-04-22

**Authors:** Patricia Gasalla, Kerrie L. Thomas, Lawrence Wilkinson, Jeremy Hall, Dominic Michael Dwyer

**Affiliations:** ^1^ Neuroscience & Mental Health Innovation Institute, School of Medicine Cardiff University Cardiff UK; ^2^ School of Psychology Cardiff University Cardiff UK

**Keywords:** anhedonia, *CACNA1C*, depression, psychosis

## Abstract

Genetic variation in *CACNA1C*, which encodes the alpha‐1 subunit of Ca_v_1.2 L‐type voltage‐gated calcium channels, is strongly linked to risk for psychiatric disorders including schizophrenia, bipolar disorder, and major depression. Here we investigated the impact of mutations of one copy of *Cacna1c* (leading to low gene dosage of *Cacna1c*) on rats' hedonic responses to palatable sucrose (assessed using the analysis of consumption microstructure). In addition, we also investigated the effects of combining either juvenile or adult stress with the manipulation of *Cacna1c*. Across three experiments, *Cacna1c* ^+/−^ rats displayed attenuated hedonic reactions to sucrose compared to wild‐type littermate controls, despite the *Cacna1c* ^+/−^ rats retaining sensitivity to sucrose concentration in terms of the amount of consumption. Unexpectedly, juvenile stress enhanced rather than reduced hedonic reactions to sucrose, while adult stress did not have clear hedonic effects. The effects of *Cacna1c* manipulation did not interact with either juvenile or adult stress. The fact that *Cacna1c* ^+/−^ rats display a clear analogue of anhedonia—a reduction in the positive hedonic reactions normally elicited by highly palatable sucrose—a symptom observed trans‐diagnostically across psychiatric disorders linked to *CACNA1C*, suggests this model may play a valuable role in the translational investigation of anhedonia.

## Graphical abstract

*CACNA1C* is linked to risk for psychiatric disorders where anhedonia is a symptom, thus we examined hedonic reactions to sucrose in a rat model of low‐dose Cacna1c. Male and female *Cacna1c* rats showed anhedonic reactions to sucrose (as lower lick cluster sizes) that did not interact with stress effects.

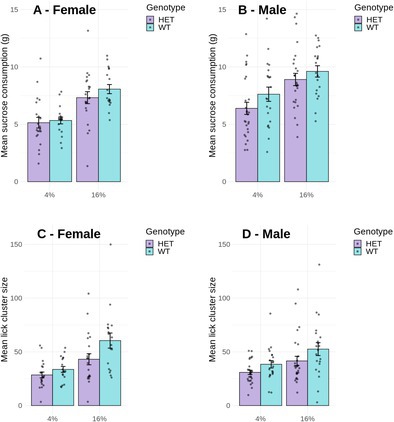

## 1 Introduction

Schizophrenia (SCZ) and bipolar disorder (BD), which can both exhibit psychotic features, are severe adult psychiatric disorders affecting approximately 3% of the population [1, 2, 3]. Both are highly heritable [4, 5] with a large degree of overlap of genetic risk between them (as well as between both and major depression) [6]. Although the psychotic symptoms (e.g., delusions, hallucinations) of SCZ are relatively amenable to pharmacological treatment, the negative symptoms (e.g., anhedonia, amotivation) are not, and these are strongly associated with functional impairment and remain poorly understood mechanistically [3, 7]. Similarly, reasonable acute pharmacological treatments for mania in BD exist but with risk of exacerbating depressive symptoms, and antidepressants can risk exacerbating mania [1]. Thus, there is a clear unmet need for understanding the biological basis for the negative symptom of psychosis.

Genomic studies of SCZ and BD have demonstrated the importance of genetic variation in voltage‐gated calcium channels (VGCCs) in risk for these conditions [8, 9]. VGCCs play a critical role in regulating calcium influx and synaptic plasticity in the central nervous system [10]. Single nucleotide polymorphism (SNP) variants in *CACNA1C*, which encodes the pore‐containing α1 subunit of the L‐Type VGCC Ca_v_1.2, have strong and replicated genome‐wide association with both SCZ [9, 11] and BD [8, 12]. Moreover, altered dosage of *CACNA1C* is important for risk, with evidence suggesting a particular role for low dosage in limbic regions [13, 14, 15], but see also [16]. In light of the transdiagnostic display of symptoms such as anhedonia, it is notable that genetic variation in VGCCs is also associated with risk for MDD [17, 18].

The investigation of *CACNA1C* contributions to the risk for neuropsychiatric disease has been furthered by the development of a rat model which reflects altered dosage seen in SCZ and BD [14]. Prior characterization of these *Cacna1c* ^+/−^ rats (with 4 bp deletion in exon 6) confirms reduced *Cacna1c* mRNA and Ca_v_1.2 α1 subunit protein expression between 17% and 48% depending on brain region [19, 20]. These rats also display aberrant Ca^2+^ signaling and downregulation of the ERK pathway in the hippocampus, as well as impaired latent inhibition [21]. Activation of the ERK pathway with a BDNF mimetic rescued synaptic plasticity and attenuated latent inhibition deficits characteristic of cognitive dysfunction observed in psychosis [21]. However, cognitive deficits are only a subset of the relevant symptoms, and reward processing deficits central to the negative or affective symptom clusters observed trans‐diagnostically remain under‐investigated, especially in relation to *Cacna1c* gene dosage.

Importantly, negative symptoms are not monolithic, reflecting separable dysfunctions in reward processing: including direct hedonic deficits (anhedonia), motivational problems (amotivation), and reward‐related cognitive biases—with variation in the presentation of symptoms across individuals. Here, we investigate a rodent analogue of anhedonia, or the reduction in positive hedonic reactions typically elicited by positive stimulation, by examining the microstructure of consumption behavior. Rodents typically produce clusters of licks separated by pauses, and the mean number of licks per cluster displays a positive monotonic relationship with the concentration of palatable sucrose solutions [22, 23, 24], a negative relationship with unpalatable solutions such as quinine [25, 26], as well as being sensitive to pharmacological interventions known to affect hedonic reactions in humans [27, 28]. Critically, lick cluster size is not simply a proxy for consumption: studies of conditioned taste aversion and preference have also shown that palatability and consumption can dissociate [29, 30, 31, 32, 33]. In the present experiments, we used the analysis of lick cluster size to provide a means of selectively assessing hedonic responses. In this light, a reduction in lick cluster size compared to control animals while consuming highly palatable sucrose solution is a clear analogue of anhedonic reactions [34, 35].

In addition to genetics, environmental risk factors are also known to play an important role in the development of psychotic illnesses [3, 36]. Stress, in both adulthood [37] and childhood [38], is linked to psychiatric diseases. The *CACNA1C* gene, specifically the rs1006737 A risk allele, is associated with a reduced cortisol awakening response in adults exposed to early life stress, indicating altered stress regulation [39]. This aligns with the theory that this *CACNA1C* risk allele impacts the stress response [40], partly because stress‐induced increases in calcium influx through Ca_v_1.2 channels are critical for the normalization of the neuronal stress response [41]. There is an interaction of adult trauma and other common *CACNA1C* risk variants with depressive symptoms [42]. Pre‐pubertal stress in rats leads to anxiety‐like behavior and reduced *Cacna1c* expression in adulthood [43, 44, 45]. Chronic social stress in adulthood increases the susceptibility to developing anxiety‐like behaviors in *Cacna1c* heterozygous mice and lowers *Cacna1c* expression in WT [42, 46]. These results suggest that genetic and environmental risk factors for psychosis may converge on VGCCs, through altered expression of *CACNA1C*. However, neither the impact of this juvenile (or adult) stress procedure on hedonic behavior, nor its potential interaction with direct manipulation of *Cacna1c* has been investigated. Therefore, in addition to examining the effect of low‐dose *Cacna1c* on hedonic behavior using the heterozygous *Cacna1c* ^+/−^ rat model (Experiment 1), Experiment 2 combined this manipulation with the effects of juvenile stress (JS), while Experiment 3 examined the effects of the same stress protocol delivered in adult *Cacna1c* ^+/−^ rats.

## 2 Materials and Methods

### 2.1 Subjects

*Cacna1c* hemizygous (*Cacna1c* ^+/−^) rats (HET) on a Sprague Dawley background (TGR16930, Horizon, Sage Research Labs, USA) and wild‐type (WT) littermates were bred at Cardiff University, UK (for further details of the model see [19, 20]). Animals were housed in single‐sex and mixed‐genotype groups of two to three in standard cages (38 × 56 × 22 cm) under a 12/12 h light/dark cycle. The housing conditions included poplar bedding (Datesand, UK) and enrichment items in the home cage. For enrichment, each cage was equipped with a rat tunnel (125 × 90 × 5 mm) and aspen wood bricks (10 × 2 × 2 cm, rt. chew sticks) (Datesand, UK). Prior to the start of each experiment, rats were moved to a food deprivation schedule with daily access to food to maintain animals between 85% and 90% of their ad lib weights. Animals had access to ad lib water throughout the experimental sessions. All experimental manipulations took place during the light phase of the cycle.

Experiment 1 used both female and male WT and HET rats: 21 HET female, 18 WT female, 29 HET male, and 22 WT male at approximately 10 weeks old. The weight range at the beginning of the experiment for females was 194–251 g, and for males it was 328–488 g. Experiment 2 used two cohorts of animals: in the JS groups, there were 13 HET females (4/9 in cohort one and two, respectively), 18 HET males (12/6), 17 WT females (12/5), and 19 WT males (9/10); in the Control groups (non‐stressed littermates that were just weighed and handled on same days as the stress animals), there were 17 HET females (11/6), 18 HET males (10/8), 11 WT females (4/7), and 11 WT males (9/2). The weight range at the beginning of the experiment for females was 180–268 g, and for males it was 222–306 g. Experiment 3 also used two cohorts of animals: in the Adult Stress (AS) groups, there were 13 HET females (6/7 animals in cohort one and two, respectively), 13 (8/5) HET males, 12 (5/7) WT females, and 15(9/6) WT males; in the Control groups (non‐stressed littermates that were just weighed and handled on same days as the stress animals), there were 12 (6/6) HET females, 11 (7/4) HET males, 9 (5/4) WT females, and 18 (8/10) WT males. The weight range at the beginning of the experiment for females was 222–306 g, and for males it was 327–505 g. In no experiment was there a significant difference between HET and WT animals in their preexperiment weights.

All experiments were conducted in accordance with local ethics guidelines, the UK Animals (Scientific Procedures) Act 1986 (PPL P0EA855DA held by Jeremy Hall) and the European Communities Council Directive (1986/609/EEC).

### 2.2 Stimulus and Apparatus

All experiments used 4%, 8%, and 16% (w/w) sucrose solutions made with deionized water. Training and testing phases took place in a room containing six identical conditioning boxes (38 × 24 × 21 cm: height × width × depth; Med Associates). The side walls of the boxes were constructed from aluminum, whereas the front, back, and the ceiling were made from clear acrylic. The floor was formed from 19 steel rods (4.8 mm diameter, 16 mm apart) placed above a stainless‐steel tray. One aluminum wall contained two 1 cm diameter holes, one at the left and one at the right side, each 5 cm from the respective wall and from the floor of the box. These holes allowed for drinking bottles consisting of a steel spout and 50 mL bottle to be accessed by rats inside the box, while the bottles were in the forward position. Bottles were automatically advanced/withdrawn at the beginning/end of each session. Licks were recorded by a PC running MEDPC (Med Associates, St Albans), which measured contacts with a bottle spout to the nearest 0.01 s. Bottles were weighed with a scale accurate to 0.01 g before and after each session. A weighing boat was placed below the hole and outside the box to collect any spill produced by retracting the bottle, ensuring accuracy in the consumption measure.

### 2.3 Procedures

#### 2.3.1 Consumption Behavior Experiment 1

At the beginning of the experiment, animals were moved to a food restriction schedule until they reached 90% of ad libitum body weight. Training started 4 days after being moved to food restriction. The training phase consisted of 10 sessions in which animals received 10 min of access to 8% (w/w) sucrose solution daily in order to habituate them to the boxes in the experimental room, timing, and procedures. Lick cluster size and consumption were measured for each animal in each session. Drinking sessions took place between approximately 9 a.m. and 2 p.m. every day (with the order counterbalanced across HET/WT and male/female animals) and rats received a measured food ration in their home cages in the afternoon. Once all animals displayed stable consumption and licking patterns, they were moved to the test phase. Half of the animals were presented with 4% (w/w) sucrose solution for 10 min daily for four consecutive days, while the other half were presented with 16% sucrose solution. Animals were then presented with the alternative solution across another four daily sessions. This means that the animals that previously received 4% sucrose solution were administered the 16% sucrose solution, and vice versa.

#### 2.3.2 Juvenile Stress Experiment 2

Between postnatal days 25 and 27, half of the animals in Experiment 2 underwent a short‐term mild stress protocol, adapted from that used in our laboratory [43, 44, 47, 48]. The animals were moved to an experimental room different from the holding room and sucrose test room. Sessions started at 10 a.m. and finished by approximately 3 p.m. On postnatal day 25, animals were subject to a 10 min forced swim session inside an opaque swimming tank measuring 25 cm in height and 34 cm in diameter. The tank, with a capacity of 12 L, contained approximately 6 L of water at a consistent temperature of 25°C ± 1°C. Animals were released facing the wall of the tank and retrieved after 10 min. Immediately after retrieval, the rats were dried with a towel and closely monitored for 30 min in the experimental room. Animals were further monitored in the home cage and did not show prolonged signs of distress following removal from the water. The following day (postnatal day 26), animals from stress groups were exposed to an elevated platform (15 × 15 cm^2^, 115 cm high) for three 30‐min sessions separated by 60 min each round. Finally, on postnatal day 27, animals were exposed to 6 × 0.5 s shocks (0.5 mA) separated by 30 s intervals. Shocks were delivered in a room containing four identical conditioning boxes (30 × 24 × 21 cm: height × width × depth; Med Associates) housed within an individual sound and light attenuating chamber. The side walls of the boxes were constructed from aluminum, whereas the front, back, and the ceiling were made from clear acrylic. The floor was formed from 19 steel rods (4.8 mm diameter, 16 mm apart) placed above a stainless‐steel tray. Illumination was provided by standard house light (40 mA) mounted in the back wall of each chamber. The electric shock was delivered using a scrambled shocker (Campden Instruments Ltd. Model HSCK1000). The rats were video monitored during the sessions. Animals were then returned to the home cage and remained undisturbed, apart from regular welfare checking and general husbandry until reaching adulthood at postnatal day 70. All animals caged together belonged to the same group (JS or Control) to avoid any potential stress transfer between animals.

Behavioral testing procedures were the same as in Experiment 1, with the exception that animals had access to 8% sucrose solution for 6 h in their home cages prior to commencing the training phase to reduce neophobic effects and promote consumption in the experimental boxes. Drinking sessions took place in the morning (approximately 10 a.m. to 3 p.m.) and rats received a measured food ration in their home cages in the afternoon.

#### 2.3.3 Adult Stress Experiment 3

The stressors used were the same as in Experiment 2, save that they were delivered once animals reached adulthood (approximately 8–9 weeks old). Stress sessions started at 10 a.m. and finished at approximately 3 p.m. A week after delivering the last stressor, the behavioral procedure began. Behavioral testing procedures were the same as in Experiment 2, with the exception that training lasted for eight sessions and testing for six sessions because steady consumption levels were reached more rapidly than in previous experiments.

### 2.4 Data Analysis

Total consumption and mean lick cluster size were the main dependent variables. Lick cluster size was defined as a group of licks separated by intervals of less than 0.5 s, a criterion that has been extensively employed in our laboratory [29, 49, 50, 51], following the original proposal by Davis [23, 52, 53]. For the tests, the average of all sessions at each sucrose concentration was calculated for each animal.

For Experiment 1, mixed ANOVA analyses were performed with concentration (4% vs. 16%) as a within‐subject variable and sex (male vs. female) and genotype (HET vs. WT) as between‐subject factors. Experiments 2 and 3 used mixed ANOVA with concentration (4% vs. 16%) as a within‐subject variable and stress group, sex, and genotype as between‐subject factors. All tests reported here used a criterion for significance of *p* = 0.05. While the order of testing can impact consumption behavior (e.g., allowing a negative contrast effect when shifting from 16% to 4% sucrose—for an example see [54]), the counterbalance of presentation order is needed to avoid confounding solution concentration with test order. Although not reported here, additional analysis of the data from all experiments with an additional between‐subject factor of test order did not impact the significance (or non‐significance) of the key factors of genotype, sex, concentration, or stress group, nor on interactions between these factors.

In all experiments, animals were excluded from the analysis if there was no lick cluster data for one or both concentrations (either because consumption was minimal or the lick recording itself failed). In Experiment 1, three animals (one WT male, two HET males) were excluded; in Experiment 2, two animals (both HET control male) were excluded; and in Experiment 3, nine animals (two WT stress males, two WT control males, two HET stress males, one WT stress female, one HET control male, and one HET control female) were excluded. Data for all experiments can be found at the OSF on https://osf.io/z4dvp/?view_only=756df66a0db1447ea94624bb27c04095.

## 3 Results

### 3.1 Experiment 1

**FIGURE 1:**
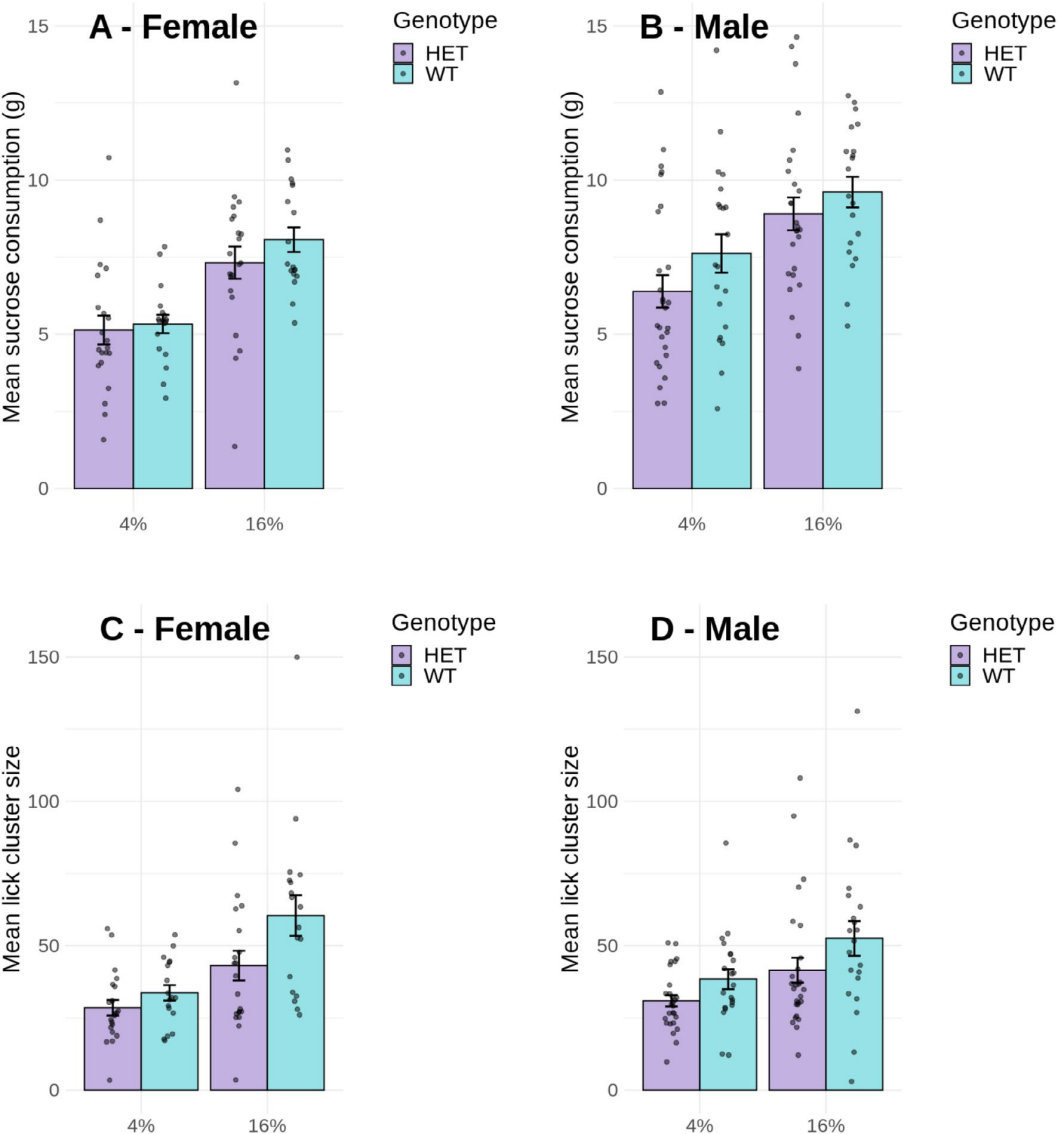
Average test data (over four sessions) for female and male HET and WT animals. Test duration was 10 min where animals had ad lib access to either a 4% sucrose solution or a 16% sucrose solution on different days. (A and B) Mean sucrose consumption in grams for females and males, respectively, and (C and D) mean lick cluster size displayed by female and male animals, respectively. Error bars represent the standard errors of the mean (SEM), and individual animals are shown as jittered dots. ANOVA analysis of consumption revealed significant main effects of concentration and sex, but no significant effect of genotype nor any interaction. The same analysis of lick cluster size revealed significant main effects of genotype and concentration, but no significant effect of sex nor any interaction (see Section 3.1 for details of the inferential analysis).

Figure 1 shows the consumption (Panels A—female, and B—male) and lick cluster size (Panels C—female, and D—male) data over test sessions. Mixed ANOVA performed on the consumption data during the test revealed significant main effects of solution concentration, *F*(1,83) = 114.47, *p* < 0.001, MSE = 2.31, *ƞ* ^2^ = 0.58, and sex, *F*(1,83) = 14.23, *p* < 0.001, MSE = 9.73, *ƞ* ^2^ = 0.15. However, the analysis revealed no significant effect of genotype, *F*(1,83) = 2.26, *p* = 0.137, MSE = 9.73, *ƞ* ^2^ = 0.01, nor any significant interaction between factors (largest *F* for concentration by sex by genotype interaction, *F*(1,83) = 1.34, *p* = 0.251, MSE = 2.31, *ƞ* ^2^ = 0.02). While males consumed more sucrose than females, there was no effect of genotype, and both HET and WT animals had similar consumption levels. The fact that the consumption of 16% sucrose was higher than that of 4% sucrose for both genotypes indicates both HET and WT animals were sensitive to sucrose concentration and showed a preference for higher sucrose concentrations.

The same analysis performed on the palatability data revealed significant main effects of solution concentration, *F*(1,83) = 50.10, *p* < 0.001, MSE = 232.68, *ƞ* ^2^ = 0.38, and importantly, of genotype, *F*(1,83) = 7.76, *p* = 0.007, MSE = 578.26, *ƞ* ^2^ = 0.09. There was no significant effect of sex, *F*(1,83) = 0.35, *p* = 0.554, MSE = 578.26, *ƞ* ^2^ < 0.01, nor any significant interaction between factors (largest *F* for concentration by sex interaction, *F*(1,83) = 3.18, *p* = 0.078, MSE = 232.68, *ƞ* ^2^ = 0.04). These results showed that HET animals had a lower lick cluster size than WT, regardless of solution concentration and sex. Thus, there was clearly an analogue of anhedonia in the *Cacna1c* HET animals that cannot be explained as a failure to detect differences in sucrose concentration given that HET animals did show concentration effects in the amount of sucrose consumed.

### 3.2 Experiment 2

**FIGURE 2:**
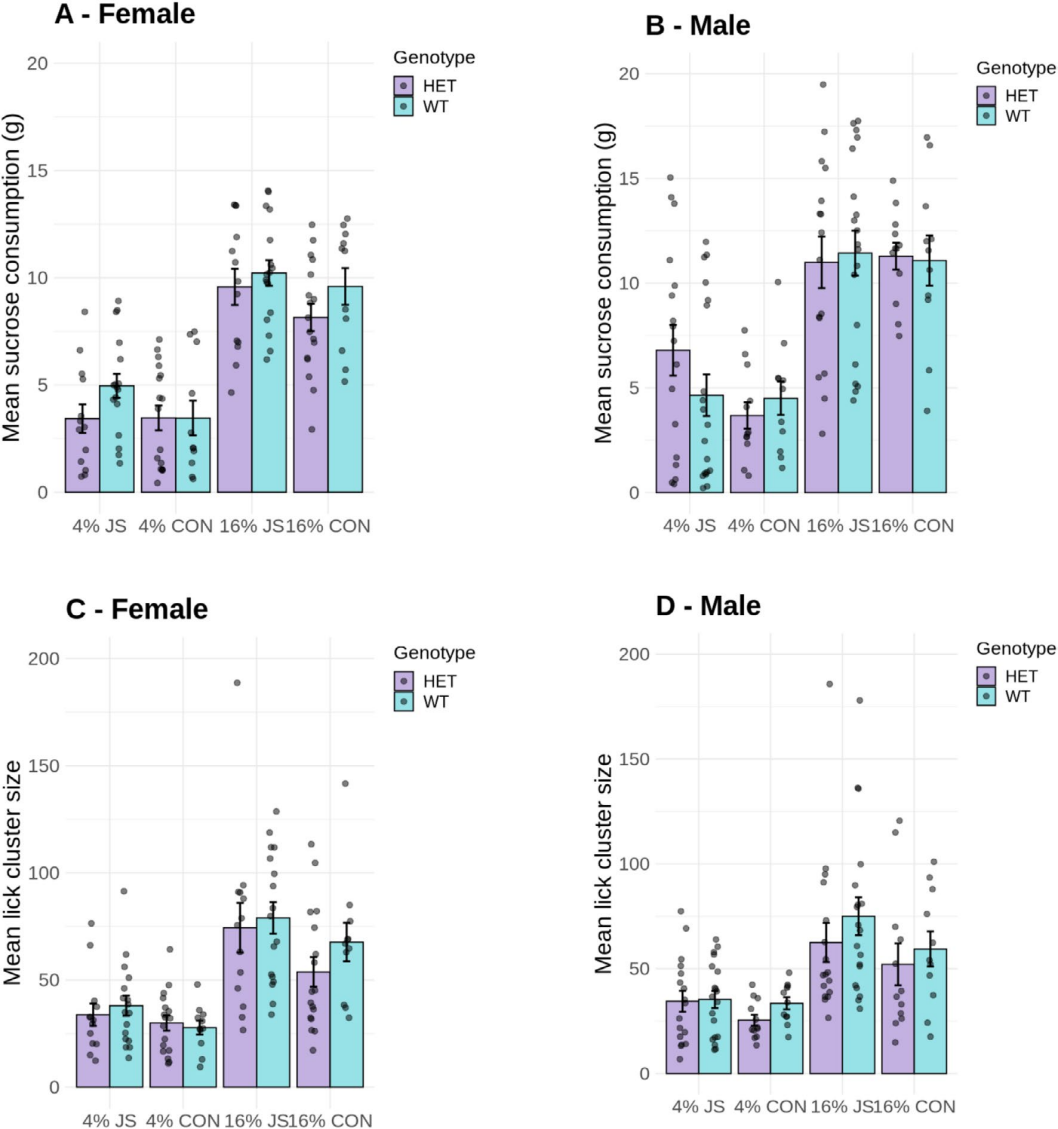
Average test data (over four sessions) for female and male HET and WT animals as a function of juvenile stress exposure (JS) versus control (CNT). Test duration was 10 min where animals had ad lib access to either a 4% sucrose solution or a 16% sucrose solution on different days. (A and B) Mean sucrose consumption in grams for females and males, respectively, and (C and D) mean lick cluster size displayed by female and male animals, respectively. Error bars represent the standard errors of the mean (SEM), and individual animals are shown as jittered dots. ANOVA analysis of consumption revealed significant main effects of concentration and sex, but no other significant main effects or interactions. The same analysis of lick cluster size revealed a marginally significant main effect of genotype, as well as main effects of concentration and stress group, but no other significant main effects or interactions (see Section 3.2 for details of the inferential analysis).

Figure 2A,B shows the mean sucrose consumption in grams for females and males, respectively. A mixed ANOVA performed with the consumption data revealed significant main effects of solution concentration, *F*(1,114) = 366.70, *p* < 0.001, MSE = 5.43, *ƞ* ^2^ = 0.76, and sex, *F*(1,114) = 7.13, *p* = 0.009, MSE = 18.93, *ƞ* ^2^ = 0.06. However, the analysis revealed no significant main effects of genotype, *F*(1,114) = 0.18, *p* = 0.673, MSE = 18.93, *ƞ* ^2^ < 0.01, or stress, *F*(1,114) = 2.56, *p* = 0.113, MSE = 18.93, *ƞ* ^2^ < 0.01, nor any significant interaction between factors (largest *F* for concentration by sex by genotype by stress interaction, *F*(1,114) = 1.80, *p* = 0.157, MSE = 5.43, *ƞ* ^2^ < 0.01). These results are consistent with Experiment 1. While males consumed more sucrose than females, there was no genotype or stress effect on the amount of sucrose consumed. Again, the higher consumption of 16% sucrose than 4% sucrose indicates that all groups were sensitive to sucrose concentration and showed a preference for higher concentrations.

Figure 2C,D shows the mean lick cluster size for females and males, respectively. Analysis of the palatability data revealed a significant main effect of solution concentration, *F*(1,114) = 106.36, *p* < 0.001, MSE = 752.48, *ƞ* ^2^ = 0.48, and a marginally significant effect of genotype, *F*(1,114) = 3.55, *p* = 0.062, MSE = 1102.22, *ƞ* ^2^ = 0.03, as well as a significant effect of stress group *F*(1,114) = 7.36, *p* = 0.008, MSE = 1102.22, *ƞ* ^2^ = 0.06. Animals display higher lick cluster sizes for the higher sucrose concentration, but more importantly, HET animals displayed a similar pattern to Experiment 1 of lower lick cluster sizes compared to WT, regardless of the sex of the animal and previous stress experience (albeit this effect was only marginally significant in the current experiment). In addition, rats that experienced JS displayed higher lick cluster sizes than control animals, suggesting higher palatability for sucrose. The remainder of the analysis revealed no significant effect of sex *F*(1,114) = 1.15, *p* = 0.286, MSE = 1102.22, *ƞ* ^2^ < 0.01, nor any significant interaction between factors (largest *F* for concentration by genotype interaction, *F*(1,114) = 1.92, *p* = 0.169, MSE = 752.48, *ƞ* ^2^ = 0.02).

Experiment 2 generally replicated the effects of direct *Cacna1c* manipulation seen in Experiment 1, namely that the behavior of HET animals was consistent with a reduction in hedonic reactions to sucrose compared to WT. However, while there was also a clear effect of stress group, it was in the opposite direction to our expectations—rats subject to JS displayed higher hedonic reactions to sucrose as adults compared to controls. These two effects appeared to be independent of each other.

### 3.3 Experiment 3

**FIGURE 3:**
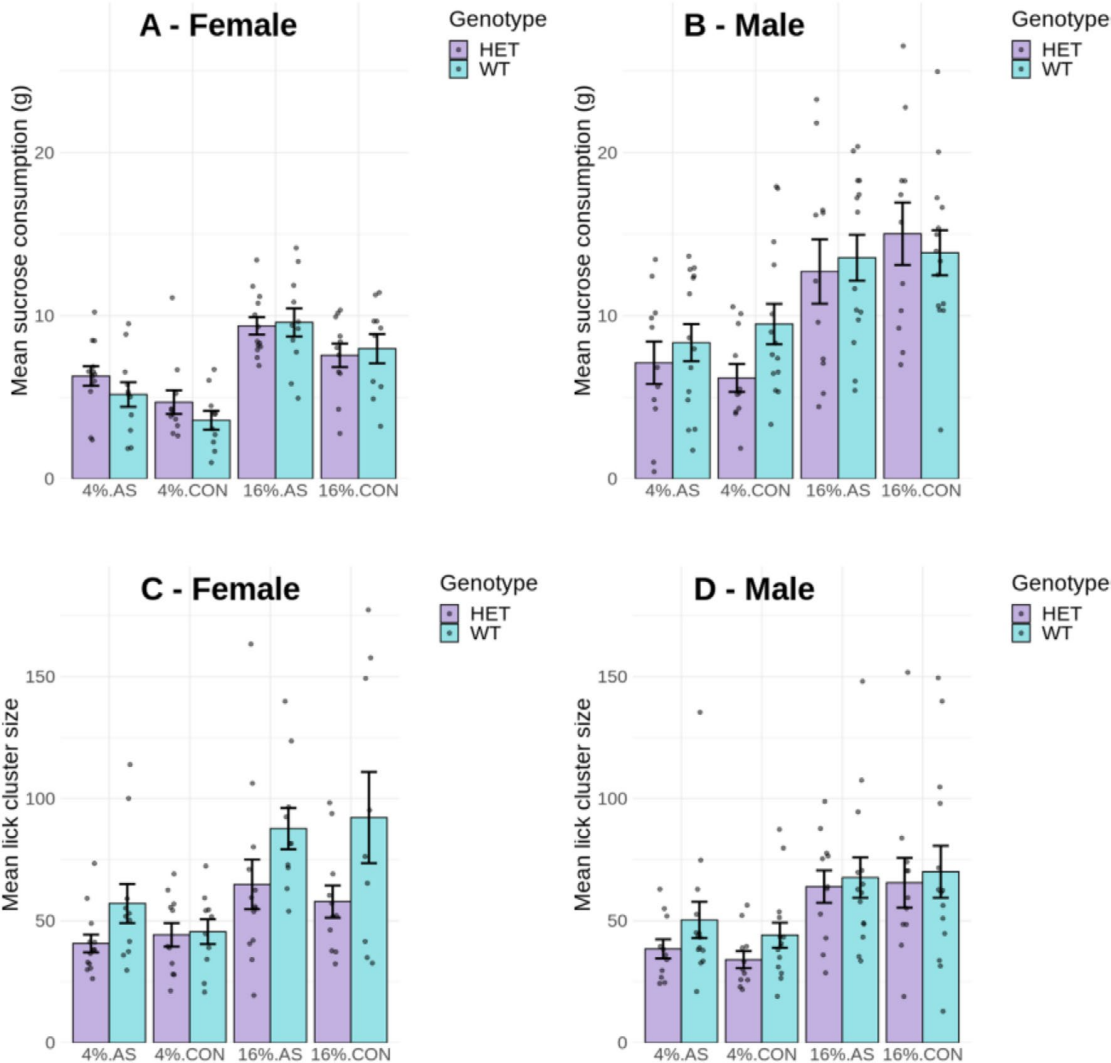
Average test data (over three sessions) for female and male HET and WT animals as a function of adult stress exposure (AS) versus control (CNT). Test duration was 10 min, where animals had ad lib access to either a 4% sucrose solution or a 16% sucrose solution on different days. (A and B) Mean sucrose consumption in grams for females and males, respectively, and (C and D) mean lick cluster size displayed by female and male animals, respectively. Error bars represent the standard errors of the mean (SEM), and individual animals are shown as jittered dots. ANOVA analysis of consumption revealed significant main effects of concentration and sex, as well as significant sex by concentration, and sex by concentration by genotype interactions (simple effect analyses revealing a significant difference between male HET and WT animals for 4% sucrose but no other significant HET/WT differences). There were no other significant main effects or interactions. The same analysis of lick cluster size revealed significant main effects of genotype and concentration, as well as a significant genotype by concentration by sex interaction (simple effect analyses revealing a significant difference between male HET and WT animals for 4% sucrose, and a significant difference between female HET and WT animals for 16% sucrose, but no other significant HET/WT differences). There were no other significant main effects or interactions (see Section 3.3 for details of the inferential analysis).

Figure 3A,B shows the mean sucrose consumption in grams for females and males, respectively. As in previous experiments, consumption was higher for 16% than for 4% sucrose and higher for males than for females. In addition, the male/female difference in consumption appeared to be reduced in animals subject to AS. A mixed ANOVA performed with the consumption data revealed significant main effects of solution concentration, *F*(1,86) = 171.97, *p* < 0.001, MSE = 6.33, *ƞ* ^2^ = 0.667, and sex, *F*(1,86) = 27.48, *p* < 0.001, MSE = 25.77, *ƞ* ^2^ = 0.24, as well as a significant sex by concentration interaction, *F*(1,86) = 9.69, *p* = 0.003, MSE = 6.33, *ƞ* ^2^ = 0.10, and a significant sex by concentration by genotype interaction, *F*(1,86) = 6.90, *p* = 0.010, MSE = 6.33, *ƞ* ^2^ = 0.07. Follow‐up analysis of this three‐way interaction revealed that male HET animals consumed less 4% sucrose than their WT littermates, *F*(1,86) = 5.60, *p* = 0.020, MSE = 20.74, *ƞ* ^2^ = 0.061, but there were no other HET/WT differences (largest *F*(1,86) = 0.88, *p* = 0.352, MSE = 11.36, *ƞ* ^2^ = 0.01 for the comparison in female animals for 4% sucrose). However, the analysis revealed no main differences between genotype, *F*(1,86) = 0.31, *p* = 0.581, MSE = 25.77, *ƞ* ^2^ < 0.01, stress group, *F*(1,86) = 0.56, *p* = 0.457, MSE = 25.77, *ƞ* ^2^ = 0.01, nor any other significant interaction between factors (largest *F* for stress group by sex interaction, *F*(1,86) = 2.88, *p* = 0.093, MSE = 25.77, *ƞ* ^2^ = 0.03).

Figure 3C,D shows the mean lick cluster size for females and males, respectively. Contrary to JS, there was no suggestion that AS enhanced lick cluster size; however, we replicated the previously observed reduction in lick cluster size for *Cacna1c* rats. ANOVA revealed a significant main effect of solution concentration, *F*(1,86) = 67.53, *p* < 0.001, MSE = 690.75, *ƞ* ^2^ = 0.44, a main effect of genotype, *F*(1,86) = 8.29, *p* = 0.005, MSE = 1588.78, *ƞ* ^2^ = 0.09, and a significant genotype by concentration by sex interaction *F*(1,86) = 8.84, *p* = 0.004, MSE = 690.75, *ƞ* ^2^ = 0.09. Follow‐up analysis of this interaction revealed that there was a significant difference between HET and WT female animals for 16% sucrose, *F*(1,86) = 11.81, *p* < 0.001, MSE = 1932.70, *ƞ* ^2^ = 0.12, but not for 4% sucrose, *F*(1,86) = 1.40, *p* = 0.241, MSE = 346.83, *ƞ* ^2^ = 0.02, as well as a significant difference between HET and WT male animals for 4% sucrose, *F*(1,86) = 4.26, *p* = 0.042, MSE = 346.83, *ƞ* ^2^ = 0.05, but not for 16% sucrose, *F*(1,86) = 0.11, *p* = 0.743, MSE = 1932.70, *ƞ* ^2^ = 0.01. In addition, there were no significant effects of stress group, *F*(1,86) = 0.33, *p* = 0.566, MSE = 1588.78, *ƞ* ^2^ < 0.01, or sex, *F*(1,36) = 3.37, *p* = 0.070, MSE = 1588.78, *ƞ* ^2^ = 0.04, nor any other interaction (largest *F* for the concentration by sex interaction, *F*(1,86) = 3.00, *p* = 0.087, MSE = 690.75, *ƞ* ^2^ = 0.03).

Thus, unlike in Experiment 2 with JS, there was no clear enhancement of palatability reactions following AS. In addition, the general pattern of lower hedonic reactions for the *Cacna1c* HET rats was clearly replicated here, albeit the genotype effect was more prominent for female rats at high sucrose concentration, but more prominent for male rats at low sucrose concentration. This variability in the overall effect of genotype across test solution concentration and sex was not clearly present in either of Experiments 1 and 2, suggesting there may be non‐systematic variation in the size of the genotype effect across experimental conditions.

## 4 Discussion

The most clear and consistent result across Experiments 1, 2, and 3 is that *Cacna1c* ^+/−^ rats displayed lower lick cluster sizes when consuming sucrose than WT littermate rats, indicating a clear effect of the *Cacna1c* genotype on hedonic responses. This effect was present in both male and female rats and did not interact with either juvenile or AS. However, the size of the effect did vary, with the main effect of genotype only marginally significant in Experiment 2, and a sex by concentration by genotype interaction was observed in Experiment 3. There was no clear pattern to this variation in effect size and so it would be premature to speculate on its cause, save to note that further experimental work is currently being undertaken that will hopefully speak to this issue. Importantly, this effect on lick cluster size was present despite the fact that *Cacna1c* ^+/−^ rats remained sensitive to differences in sucrose concentration in terms of its impact on the overall amount of sucrose consumed. Thus, the lick analysis suggests the presence of a true hedonic deficit that cannot be reduced to a failure to detect sucrose itself. In short, *Cacna1c* ^+/−^ rats display a clear analogue of anhedonia—they show a reduction in the positive hedonic reactions normally elicited by highly palatable sucrose. In addition to this genotype effect, JS unexpectedly resulted in an increase in hedonic reactions to sucrose (Experiment 2), while a similar effect was not observed after AS (Experiment 3). These results have implications for both the investigation of the biological mechanisms contributing to anhedonia and for the understanding of stress and resilience. We will cover each of these issues in turn.

Considered across all experiments, the general observation of a defect in hedonic reactions to sucrose in *Cacna1c* ^+/−^ rats is not only consistent with the fact that variation in this VGCC‐encoding gene is associated with risk for multiple psychiatric disorders where anhedonia is a key symptom, but also consistent with the specific impacts of variation in *CACNA1C* in humans on reward processing. For example, using a probabilistic reward‐learning task, Lancaster and colleagues [55] found that individuals with the A risk allele carriers (AA/AG) of the *CACNA1C* rs1006737 genotype had a deficit in reward responsiveness compared with the non‐risk (GG) genotype group. In addition, the amount of amygdala activation in response to a reward reversal learning task also differed between the A risk allele carriers (AA/AG) of rs1006737 and the non‐risk (GG) genotype group [56]. Moreover, A risk allele carriers of rs1006737 also displayed altered resting‐state functional connectivity across a network of brain regions, including those associated with emotion and reward processing [57]. Taken together with the current results, these studies of *CACNA1C* variation in humans suggest the presence of a translationally preserved deficit in the behavioral responses to rewards and in the function of reward‐related brain networks. Overall, these results support the view that genetic variation in *CACNA1C* may contribute to anhedonia trans‐diagnostically across a range of psychiatric presentations.

Turning to the effects of stress, we had expected that stress would be likely to produce a reduction in hedonic responses to sucrose, and for this to interact with the effect of *Cacna1c* variation. These expectations were based specifically on the fact that the JS procedure we used has been shown to impact anxiety and learning [43, 44], as well as producing a decrease in the expression of *Cacna1c* itself [45]. More generally, across a range of species and potential stressors, exposure to stress is associated with depressive behaviors [58]. Despite these expectations, JS resulted in an increase in hedonic reactions to sucrose, while AS had no significant impact on hedonic response. Although counter to our expectations, such a result is not unprecedented—a recent meta‐analytic review noted that exposure to developmental stressors had very heterogeneous effects, including a number of reports of positive effects despite the overall estimate of the effect size being negative [59]. Indeed, the fact that stress may be associated with positive outcomes in some situations is a key contributor to theoretical analyses suggesting that exposure to stress can protect against later challenges such as the inoculation‐stress hypothesis [60, 61] or the related match/mismatch hypothesis [62]. In this light, the higher hedonic reactions after JS (but not AS) could suggest that early negative experiences have produced some resilience to subsequent challenges. While intriguing, such a possibility remains speculative given that it relies on genuinely differing effects of AS and JS, and ideally, this dissociation would be replicated before firm conclusions are drawn regarding resilience effects of stress being modulated by age of experience. Regardless, the fact that there were no interactions between stress and *Cacna1c* manipulations implies that, at least in the present case, the effects of stress were not mediated via stress effects on *Cacna1c* expression.

## 5 Conclusions

Returning to the general observation that *Cacna1c* ^+/−^ rats display anhedonic reactions to sucrose, the presence of this general deficit implies that the *Cacna1c* ^+/−^ rat provides a highly valuable test bed for investigating the mechanisms by which deficiencies in VGCC function contribute to the presentation of an anhedonic phenotype. Given the fact that the *Cacna1c* ^+/−^ rat also displays attenuated latent inhibition deficits characteristic of cognitive dysfunction observed in psychosis [21], as well as deficits in fear and reversal learning [19, 20], it affords the possibility of investigating the commonalities and potential differences in the role of VGCCs across cognitive and hedonic functions. In addition, the fact that the *Cacna1c* ^+/−^ rat displays an analogue of anhedonia, a symptom observed trans‐diagnostically across many of the psychiatric disorders for which variation in *CACNA1C* presents as a risk factor, suggests this rat may play a valuable role in the translational investigation of anhedonia more generally. The fact that both some of the cognitive and synaptic plasticity deficits in the *Cacna1c* ^+/−^ rat can be rescued by activation of the ERK pathway [21] suggests an obvious target for initial investigation of the cellular mechanisms underpinning *Cacna1c*‐related hedonic deficits. Thus, drugs impacting VGCCs [63] or downstream pathways [21, 64] are of potential interest as candidate therapeutic approaches for both cognitive and hedonic deficits associated with genomic variation in *CACNA1C*.

## Conflicts of Interest

The authors declare no conflicts of interest.

## Data Availability

Data for all experiments can be found at the OSF on https://osf.io/z4dvp/?view_only=756df66a0db1447ea94624bb27c04095.
